# Safety of Cell Therapy with Mesenchymal Stromal Cells (SafeCell): A Systematic Review and Meta-Analysis of Clinical Trials

**DOI:** 10.1371/journal.pone.0047559

**Published:** 2012-10-25

**Authors:** Manoj M. Lalu, Lauralyn McIntyre, Christina Pugliese, Dean Fergusson, Brent W. Winston, John C. Marshall, John Granton, Duncan J. Stewart, Canadian Critical Care Trials Group

**Affiliations:** 1 Department of Anesthesiology, University of Ottawa, Ottawa, Canada; 2 Department of Medicine (Division of Critical Care), University of Ottawa, Ottawa, Canada; 3 Regenerative Medicine Program, The Ottawa Hospital Research Institute, Ottawa, Canada; 4 Department of Cell and Molecular Medicine, University of Ottawa, Ottawa, Canada; 5 The Ottawa Hospital Research Institute, Clinical Epidemiology Program, Ottawa, Canada; 6 Department of Critical Care Medicine, University of Calgary, Calgary, Canada; 7 Department of Surgery (Critical Care), University of Toronto, Toronto, Canada; 8 Department of Medicine (Critical Care), University of Toronto, Toronto, Canada; University of Udine, Italy

## Abstract

**Background:**

Mesenchymal stromal cells (MSCs, “adult stem cells”) have been widely used experimentally in a variety of clinical contexts. There is interest in using these cells in critical illness, however, the safety profile of these cells is not well known. We thus conducted a systematic review of clinical trials that examined the use MSCs to evaluate their safety.

**Methods and Findings:**

MEDLINE, EMBASE, and the Cochrane Central Register of Controlled Trials (to June 2011), were searched. Prospective clinical trials that used intravascular delivery of MSCs (intravenously or intra-arterially) in adult populations or mixed adult and pediatric populations were identified. Studies using differentiated MSCs or additional cell types were excluded. The primary outcome adverse events were grouped according to immediate events (acute infusional toxicity, fever), organ system complications, infection, and longer term adverse events (death, malignancy). 2347 citations were reviewed and 36 studies met inclusion criteria. A total of 1012 participants with clinical conditions of ischemic stroke, Crohn's disease, cardiomyopathy, myocardial infarction, graft versus host disease, and healthy volunteers were included. Eight studies were randomized control trials (RCTs) and enrolled 321 participants. Meta-analysis of the RCTs did not detect an association between acute infusional toxicity, organ system complications, infection, death or malignancy. There was a significant association between MSCs and transient fever.

**Conclusions:**

Based on the current clinical trials, MSC therapy appears safe. However, further larger scale controlled clinical trials with rigorous reporting of adverse events are required to further define the safety profile of MSCs.

## Introduction

Mesenchymal stromal cells (mesenchymal stem cells; MSCs) are a heterogeneous group of cells that can be isolated from many adult tissues (e.g. bone marrow, adipose tissue). First described in 1974 [1] they have recently received attention in a number of different clinical fields for their potential therapeutic effects.

Although often described as ‘adult stem cells’, MSC's have limited cellular differentiation ability. Instead, pre-clinical evidence suggests that MSCs exert their beneficial effects largely through immunomodulatory and paracrine mechanisms. MSCs home to sites of inflammation and secrete bioactive molecules, and thus may be especially effective in proinflammatory diseases. [2] There is a growing body of literature demonstrating the efficacy of MSC therapy in a variety of pre-clinical models, including acute lung injury, [3], [4] septic shock, [5] and acute myocardial infarction. [6] Several small clinical trials have investigated the efficacy and safety of MSCs in diseases including chronic heart failure, acute myocardial infarction, hematological malignancies and graft versus host disease.

There is interest in applying MSCs to pulmonary diseases (e.g. chronic obstructive pulmonary disease) and critical illness (e.g. acute respiratory distress syndrome); however, safety concerns represent a significant barrier to the successful translation of MSCs into an acceptable clinical therapeutic. These include neoplastic potential due to MSC's proliferative capacity, susceptibility to infection given their immunomodulatory effects, embolism of the cells, zoonoses associated with cell culture reagents, and acute or chronic immunogenicity of the cells themselves. [7] Therefore, we conducted a systematic review of the literature to evaluate the safety of MSC-based therapy in all clinical trials.

## Methods

### Eligibility Criteria

We included randomized and non-randomized controlled trials as well as uncontrolled clinical trials (Phase I/II trials with more than two participants) that examined the safety of intravascularly delivered MSCs in adult (at least 18 years old) or mixed adult and pediatric participants. All clinical settings were included. We excluded studies that exclusively used non-intravascular routes of administration, *ex vivo* differentiated MSCs, or co-administered MSCs with other experimental cells or treatments.

### Search Strategy

We conducted electronic searches without language restriction of Ovid MEDLINE (1950 to June 2011), EMBASE (1980 to Week 21, 2011), Cochrane Central Register of Controlled Trials (2^nd^ Quarter 2011), and the Cochrane Database of Systematic Reviews (2^nd^ Quarter 2011). Given the non-standard terminology associated with MSCs a number of terms were used (Appendix S1, search strategy). ClinicalTrials.gov was searched for ongoing or recently completed trials. Abstracts and proceedings from clinical conferences were identified and searched using Web of Science (September 2010). Bibliographies of retrieved articles and relevant reviews were searched.

### Assessment of Risk of Bias

RCTs that met inclusion criteria were assessed for risk of bias according to the Cochrane Collaboration methods. [8]

### Study Selection, Data Collection and Analysis

All study selection and data extraction was performed independently by two reviewers (M.M.L., C.P.) using standardized forms. Discrepancies were resolved by discussion with a third author (L.L.M.).

### Main Outcome Measure: Adverse Events

Adverse events were grouped according to the immediacy of the event (acute infusional toxicity, fever), the occurrence of organ system complications [neurological, pulmonary, cardiovascular (arrhythmias and other cardiac events), gastrointestinal and renal, and hematologic systems], infection, and the occurrence of longer term adverse effects (death, malignancy).

Completeness of adverse events reporting was assessed using the CONSORT approach to harm reporting. [9] Specifically, we examined whether expected adverse events were listed and defined in the methods section, whether events were described as serious versus non serious (e.g. as per Good Clinical Practice Guidelines), and if frequency and duration of follow up of adverse events was provided.

### Statistical Analysis

Meta-analyses of adverse events was performed using Comprehensive Meta-analysis (Version 2, Biostat). Data was analyzed by Peto's method with correction of zero-count cells. Pooled events were described using odds ratios (OR) and 95% confidence intervals (95% CI). An odds ratio less than 1 favoured MSC treatment. Heterogeneity between trials was evaluated using the I^2^ test [10] as well as the χ^2^ test. Sensitivity analyses were planned according to the patient population, MSC type (autologous versus allogeneic; fresh versus cryopreserved), and culture media (fetal bovine serum versus human).

Adverse events for non-randomized controlled trials with control groups that did not receive any dose of MSCs were pooled and reported according to numbers and proportions.

## Results

### Search Results

**Figure 1:**
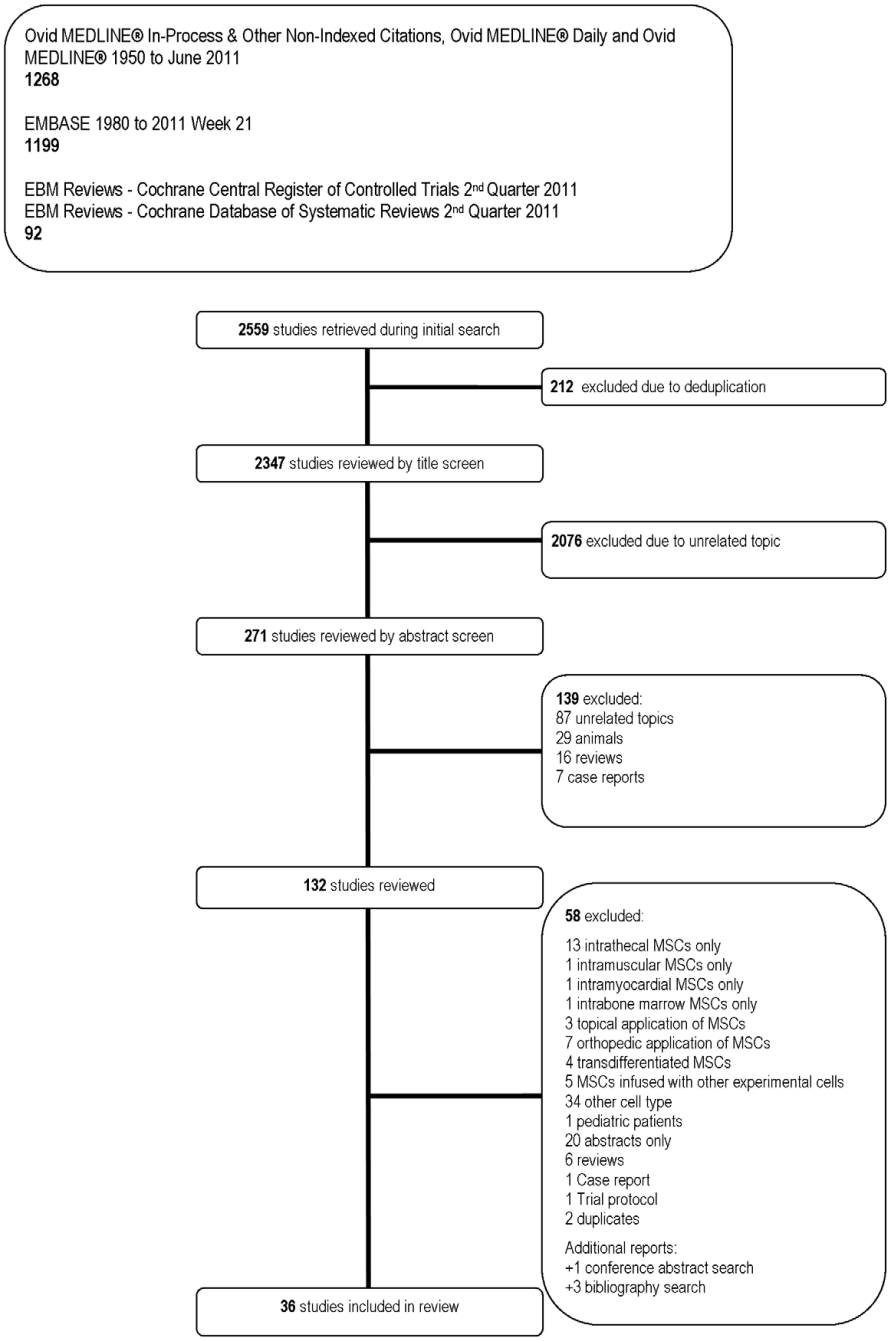
Flow Diagram of Included and Excluded Studies.

Our search identified 2347 unique titles and 36 studies met inclusion criteria (see Figure 1). Seven unpublished studies were found in a search of clinicaltrials.gov (Appendix S2). Nineteen studies were found as abstracts only (Appendix S3).

### Study Characteristics and Patient Populations

There were eight RCTs (n = 369 patients) [11]–[18] (Table 1), 10 non–RCTs (n = 466 patients) [19]–[28] (Table 2), and 18 uncontrolled clinical trials (n = 252 patients) (Table 3). [29]–[46] Six of the 36 studies were multi-centre. [13], [14], [21], [24], [33], [34] Control groups in the 10 non-RCTs were variably defined; three were prospective, [20], [26], [27] two were historical, [19], [28] and five received MSCs but in smaller doses (dose escalation design). [21]–[25] Two non-RCTs [22], [27] and one uncontrolled trial [40] had a mixed adult-pediatric population, all other studies included only adult participants. Included studies were conducted in 14 different countries from Asia, the Middle East, Europe, and North America.

**Table 1:** Characteristics of randomized control trials.

Source	Country	Patient Population	Intervention	Comparison	Patients Evaluated		Age		Follow Up Duration
		(n)	(Source, Route)		(n (%male))		(yrs±SD)		(mo)
					T	C	T	C	
**Cardiovascular**									
Hare et al, 2009 [13]	USA	acute myocardial infarction (60)	unmatched allogeneic, IV	vehicle solution, IV	34 (82)	19 (79)	59±12	55±10	6
Chen et al, 2006 [11]	PRC	ischemic heart failure (48)	autologous, IC	maximal medical therapy	22 (88)	23 (92)	59±7	57±7	12
Wang et al, 2006 [17]	PRC	idiopathic dilated cardiomyopathy (24)	autologous, IC	saline, IC	12 (75)	12 (67)	54±11	58±11	6
Chen et al, 2004 [12]	PRC	acute myocardial infarction (69)	autologous, IC	saline, IC	34 (94)	35 (97)	58±7	57±5	6
**Neurological**									
Lee et al, 2010 [14]	ROK	ischemic stroke (85)	autologous, IV	rehabilitation alone	16 (50)	36 (72)	64±12	65±15	60
Lee et al, 2008 [15]	ROK	MSA (29)	autologous, IA, IV	-	11 (82)	18 (67)	58±7	57±7	12
Xie et al, 2007 [18]	PRC	spinal cord injury (24)	autologous, IV+/−IT	rehabilitation alone	11 (81)	13 (77)	18–49*	21–53*	3
**Oncological/Hematological**									
Ning et al, 2008 [16]	PRC	stem cell transplantation for hematologic malignancy (30)	matched allogeneic, CV	stem cell transplant alone	10 (90)	15 (87)	36±11	39±12	36

Abbreviations:

− = not reported,

* = range, C = control group, CV = central venous, IA = intraarterial, IC = intracoronary, IV = peripheral intravenous, MSA = multiple systems atrophy, SD = standard deviation, PRC = People's Republic of China, RCT = randomized controlled trial, ROK = Republic of Korea, T = treatment group, USA = United States of America,

**Table 2:** Characteristics of non randomized controlled trials.

Source	Country	Patient Population (n)	Control Group	Intervention	Comparison	Patients Evaluated		Age		Follow Up Duration
				(Source, Route)		(n (%male))		(yrs±SD)		(mo)
						T	C	T	C	
**Cardiovascular**										
Mohyeddin-Bonab et al, 2007 [26]	IRN	old myocardial infarction (16)	Prospective	autologous, IC, IM	coronary occlusion alone	8 (88)	8 (75)	50±9	53±7	18
**Oncological/Hematological**										
Vanikar et al, 2011 [28]	IND	stem cell transplantation post-renal transplant (200)	Historical	autologous, OV	stem cell transplant alone	100 (82)	100 (91)	34 (8–63)+	34 (7–70)+	T: 17 (15–21) C: 26 (22–31)¥
Baron et al, 2010 [19]	BEL	stem cell transplantation for hematologic malignancy (36)	Historical	umatched allogeneic, IV	historical control, stem cell transplant alone	20 (70)	16 (81)	58 (21–69) ¥	55 (10–69)¥	30
Gonzalo-Daganzo et al, 2009 [20]	SPA	stem cell transplantation for hematologic malignancy (55)	Prospective	matched and unmatched allogeneic, IV	stem cell transplant alone	9 (56)	46 (63)	32 (21–48) ¥	35 (16–60) ¥	T: 7.4 (1–22) C: 10.3 (1–107)¥
Kebriaei et al, 2009 [21]	USA	GVHD (32)	No**	unmatched allogeneic, IV	-	L: 15 (67) H: 16 (69)	−	L: 53 (42–67)¥ H: 49 (34–67)¥	−	3
Ringden et al, 2006 [27]	SWE	GVHD (24)	Prospective	matched and unmatched allogeneic, IV	standard GVHD treatment	8 (88)	16 (56)	58 (8–61)¥	40 (3–60)¥	2–39
Lazarus et al, 2005 [24]	USA,ITL	stem cell transplantation for hematologic malignancy (56)	No**	matched allogeneic, IV	-	46 (52)# L: 20 M: 21 H: 5	-	45 (19–61)¥	-	48
Koç et al, 2002 [22]	USA	MLD and Hurler syndrome (12)	No**	matched allogeneic, IV	-	L: 4 (0) M: 3 (33) H: 4 (75)	-	L: 16±7 M: 13±10 H: 10±8	-	24 (15–31)¥
Lazarus et al, 1995 [23]	USA	volunteers with hematological malignancy in remission (23)	No**	autologous, IV	-	L: 5 (40) M: 5 (40) H: 5 (60)	-	L: 43±11 M: 37±13 H: 41±9	-	2 weeks
**Volunteer**										
Liu et al, 2006 [25]	PRC	volunteers (12)	No**	autologous, IV	-	L: 4 (75) M: 4 (75) H: 4 (75)	-	L: 32±3 M: 30±5 H: 33±3	-	3

Abbreviations:

− = not applicable, BEL = Belgium, C = control group, GVHD = graft-versus-host disease, H = high dose, IC = intracoronary, IND = India, IRN = Iran, ITL = Italy, IV = peripheral intravenous, IM = intramyocardial, L = low dose, M = middle dose, SD = standard deviation, OV = omental vein, PRC = People's Republic of China, SPA = Spain, SWE = Sweden, T = treatment group, USA = United States of America,

+ = mean (range),

¥ = median (range),

# = % male only provided for combined dose cohorts.

** Control groups received lower doses of MSCs.

**Table 3:** Characteristics of clinical trials with no control group.

Source	Country	Patient Population	Source and Route of Cells	Patients Evaluated	Age	Follow Up Duration
		(n)		(n (%male))	(yrs±SD)	(mo)
**Cardiovascular**						
Yang et al, 2010 [44]	PRC	acute myocardial infarction (16)	autologous, IC	16 (NR)	60±5	6
**Neurological**						
Honmou et al, 2011 [32]	JPN	ischemic stroke (16)	autologous, IV	12 (75)	59±8	12
Karussis et al, 2010 [33]	ISR, GRE	MS (15) ALS (19)	autologous, IT+/−IV	MS: 15 (47) ALS: 19 (52)	35±9 53±11	6–25+
Zhang et al, 2008 [46]	PRC	traumatic brain injury (7)	autologous, IV+ICN	7 (86)	39±18	6
**Oncological/Hematological**						
Wang et al, 2011 [42]	PRC	polymyositis (6) dermatomyositis (4)	umatched allogeneic, IV	10 (10)	33±10	17 (7–25)*
Arima et al, 2010 [29]	JPN	GVHD (3)	matched and unmatched allogeneic, IA	3 (33)	48±14	6
Liang et al, 2010 [35]	PRC, USA	SLE (15)	unmatched allogeneic, IV	15 (7)	28±11	17 (3–36)¥
Sun et al, 2010 [41]	PRC, USA	SLE (16)	unmatched allogeneic, IV	16 (13)	32±11	8 (3–28)¥
Weng et al, 2010 [43]	PRC	GVHD (19)	matched and unmatched allogeneic, IV	19 (74)	29±6	33 (8–92)*
Zhang et al, 2010 [45]	PRC	stem cell transplantation for hematologic malignancy (14)	matched allogeneic, IV	12 (67)	38±9	3–57+
Sun et al, 2009 [40]	PRC USA	SLE (4)	unmatched allogeneic, IV	4 (25)	19±3	12–18¥
Meuleman et al, 2009 [37]	BEL	poor hematopoietic recovery following stem cell transplantation (6)	matched allogeneic, CV	6 (100)	32±12	3
Le Blanc et al, 2008 [34]	SWE, ITL, AUS, NL	GVHD (55)	matched and unmatched allogeneic, IV	55 (62)	22 (0.5–64)*	16 (1.5–64)*
Ringden et al, 2007 [39]	SWE	pneumomediastinum (2) hemorrhagic cystits (7) perforated colon (1) following stem cell transplant	matched and unmatched allogeneic, IV	10(60)	43±19	16
Fang et al, 2007 [31]	PRC	GVHD (6)	matched and unmatched allogeneic, IV	6 (50)	39±9	17–60+
**Gastrointestinal**						
Liang et al, 2011 [36]	PRC	Crohn's disease (4) ulcerative colitis (3)	unmatched allogeneic, IV	4 (75) 3 (33)	28±3 30±10	19 (6–32)*
Duijvestein et al, 2010 [30]	NL	Crohn's disease (10)	autologous, IV	9 (22)	33±5	1.5
Mohamadnejad et al, 2007 [38]	IRN	liver cirrhosis (4)	autologous, IV	4 (25)	47±10	12

Abbreviations:

− = not applicable, ALS = amyotrophic lateral sclerosis, AUS = Australia, BEL = Belgium, C = control group, CV = central venous, GRC = Greece, GVHD = graft-versus-host disease, IA = intraarterial, ICN = intracranially, IRN = Iran, ISR = Israel, IC = intracoronary, IT = intrathecal, ITL = Italy, IV = peripheral intravenous, JPN = Japan, IM = intramyocardial, L = low dose, M = middle dose, MLD = metachromatic leukodystophy, MS = multiple sclerosis, SD = standard deviation, NL = Netherlands, NR = not reported, PRC = People's Republic of China, SLE = systemic lupus erythematosus, SWE = Sweden, USA = United States of America.

* = mean (range),

+ = range,

¥ = median (range).

Sample sizes ranged from 3 to 200 participants (28±34, mean ± standard deviation). The follow-up period was reported in all studies and the duration ranged from 0.5–60 months. Two studies (one RCT [13] and one non–RCT [21]) reported funding from a for-profit manufacturer of MSCs (Osiris Therapeutics, Inc.).

Eight RCTs included patient populations with cardiovascular disease (either acute myocardial infarction [12], [13] or chronic heart failure [11], [17]; n = 191), neurological disease (either ischemic stroke, [14] multiple systems atrophy, [15] or spinal cord injury [18]; n = 105), and following stem cell transplantation for hematological malignancies (n = 25). [16] The 10 non-RCTs included patient populations with old myocardial infarctions (n = 16), [26] stem-cell transplant post renal transplant (n = 200), [28] stem cell transplant for hematological malignancy(n = 147), [19], [20], [24] previous hematological malignancies(n = 23), [23] graft versus host disease (n = 56), [21], [27] metachromatic leukodystrophy or Hurler syndrome (n = 12), [22] or healthy volunteers (n = 12). [25] The remaining 18 uncontrolled clinical trials included patient populations with cardiovascular, neurological, oncological, and gastrointestinal disorders (n = 252).

### RCT Risk of Bias Assessment

No RCTs fulfilled all six criteria for low risk of bias (Table 4). Three trials met five of six criteria. [11]–[13] Six trials described randomization procedures with a low risk of bias. [11]–[16] Three [11]–[13] of eight studies were double blinded with one describing blinding procedures. [13] One study had an open label intervention but a blinded outcome measure. [14] Allocation concealment was performed in four of the eight RCTs. [11]–[14]

**Table 4:** Risk of bias assessment of randomized control trials.

Source	Random Sequence Allocation	Allocation Concealment	Blinding of Personnel	Blinding of Outcome Assessment	Incomplete Data Addressed	Selective Reporting
Hare et al, 2009 [13]	**L**	**L**	**L**	**L**	**H**	**L**
Chen et al, 2006 [11]	**L**	**L**	**L**	**H**	**L**	**L**
Wang et al, 2006 [17]	**U**	**H**	**H**	**H**	**L**	**L**
Chen et al, 2004 [12]	**L**	**L**	**L**	**H**	**L**	**L**
Lee et al, 2010 [14]	**L**	**L**	**H**	**L**	**L**	**L**
Lee et al, 2008 [15]	**L**	**H**	**H**	**H**	**L**	**L**
Xie et al, 2007 [18]	**U**	**H**	**H**	**H**	**L**	**L**
Ning et al, 2008 [16]	**L**	**H**	**H**	**H**	**L**	**L**

Abbreviations:

L = low risk of bias, H = high risk of bias, U = unclear risk of bias.

### MSC Preparation and Administration

Sixteen studies used autologous MSCs, [11], [12], [14], [15], [17], [18], [23], [25], [26], [28], [30], [32], [33], [38], [44], [46] eight used third party unmatched MSCs, [13], [19], [21], [35], [36], [40]–[42] five used MSCs from matched donors, [16], [22], [24], [37], [45] and seven used both matched and unmatched cells. [20], [27], [29], [31], [34], [39], [43] Twenty-seven of the 36 studies cultured the MSCs in fetal bovine serum, [11]–[17], [19]–[27], [30], [31], [33]–[35], [38]–[40], [42], [43], [46] five in human serum, [28], [29], [32], [37], [45] and four did not report the source of serum used. [18], [36], [41], [44] Nine of the 36 studies cryopreserved MSCs prior to administration [13], [19], [21], [22], [24], [30], [32], [33], [45] and one used both fresh and cryopreserved MSCs, [34] while the remainder of studies used only fresh MSCs. Fifteen investigations reported the viability of prepared MSCs (range 70–99%, median 95%). [11], [13]–[15], [17], [19]–[21], [28], [32], [34], [41], [44]–[46]

### Primary Outcome: Adverse Events

A description and frequency of adverse events is provided in Tables 5, 6, 7, 8, 9, and 10.

**Table 5:** Frequency of adverse events in randomized controlled trials.

Source	Statements of Safety and Adverse Events Reported	Frequency Treatment Group	Frequency Control Group
**Cardiovascular**			
Hare et at, 2009 [13]	Infusional toxicity	0/34	0/19
	Arrhythmia	3/34	7/19
	Organ dysfunction (cardiac)	15/34	9/19
	Organ dysfunction (gastrointestinal and renal)	9/34	4/19
	Organ dysfunction (immune)	2/34	0/19
	Infection	11/34	5/19
	Death	0/34	0/19
	Tumour/malignancy	0/34	0/19
	General disorders and administration site conditions (chest pain, fatigue)	14/34	13/19
	Rehospitalization	9/34	7/19
Chen et al, 2006 [11]	Infusional toxicity (transient pulmonary edema)	3/22	N/A
	Arrhythmia	0/22	0/23
	Death (cardiac causes)	2/22	4/23
Wang et al, 2006 [17]	Infusional toxicity (anaphylaxis, embolism)	0/12	0/12
	Fever	0/12	0/12
	Arrhythmia	0/12	0/12
	Organ dysfunction (cardiac)	1/12	0/12
	Death	1/12	2/12
Chen et al, 2004 [12]	Arrhythmia	0/34	0/35
	Death	0/34	0/35
**Neurological**			
Lee et al, 2010 [14]	Immediate:		
	Infusional toxicity (anaphylaxis)	0/16	N/A
	Fever	1/16	0/36
	Infection (pneumonia, urinary tract infection)	3/16	9/36
	Organ dysfunction (cardiac)	1/16	2/36
	Organ dysfunction (hepatic)	1/16	2/36
	Organ dysfunction (renal)	0/16	1/36
	Local complications	0/16	0/36
	Vascular obstruction (recurrent stroke)	2/16	1/36
	Vascular obstruction (peripheral artery occlusive disease)	1/16	0/36
	Late:		
	Arrhythmia	0/16	0/36
	Organ dysfunction (neurological - neuropsychological illness)	6/16	7/36
	Organ dysfunction (neurological - seizure)	3/16	5/36
	Tumour/malignancy (systemic cancer)	0/16	1/36
	Tumour/malignancy (benign mass)	1/16	1/36
	Zoonoses	0/16	0/36
	Death	4/16	21/36
Lee et al, 2008 [15]	Immediate:		
	Infusional toxicity (anaphylaxis)	0/11	N/A
	Fever	6/11	0/18
	Ischemic lesions after intraarterial injection: one small spotty lesion (<5 mm) on MRI (no neurological changes noted)	5/11	0/18
	Ischemic lesions after intraarterial injection: multiple small spotty lesions (<5 mm) on MRI (no neurological changes noted)	2/11	0/18
	Late:		
	Tumour/malignancy	0/11	0/18
	Cholecystitis (requiring cholecystectomy, patient had previous cholelithiasis)	1/11	0/18
Xie et al, 2007 [18]	Abdominal distention, anesthesia of legs and meningeal irritation – transient 2–3 days	1/11	0/13
	Fever	7/11	0/13
	Death	0/11	0/13
	Headache (patients received intrathecal MSCs)	2/11	0/13
**Hematological/Oncological**			
Ning et al, 2008 [16]	Infusional toxicity	0/10	N/A
	Infection (early/mid-phase)	4/10	5/15
	Death	6/10	5/15
	Tumour/malignancy (relapse)	6/10	3/15
	GVHD (acute)	1/10	8/15
	GVHD (chronic)	1//10	4/15

Abbreviations: AE = adverse event, FBS = fetal bovine serum, GVHD = graft versus host disease, MRI = magnetic resonance imaging.

**Table 6:** Frequency of adverse events in non randomized controlled trials.

Source	Statements of Safety and Adverse Events Reported	Frequency Treatment Group	Frequency Control Group
**Cardiovascular**			
Mohyeddin-Bonab et al, 2007 [26]	Death	0/8	0/8
**Hematological/Oncological**			
Vanikar et a, 2011 [28]	Death (all cause)	4/100	9/100
	GVHD	0/100	0/100
	Transplant rejection	0/100	6/100
Baron et al, 2010 [19]	Death (all cause, 1 year)	4/20	9/16
	Death (non-relapse, 1 year)	2/20	6/16
	Tumour/malignancy (relapse, 1 year)	6/20	4/16
	GVHD (acute)	11/20	12/16
Gonzalo-Daganzo et al, 2009 [20]	Infusional toxicity	0/9	N/A
	Death (prior to platelet recovery)	1/9	15/46
	Death (multi-organ failure without GVHD)	1/9	0/46
	Tumour/malignancy (relapse)	1/9	6/46
	GVHD (Class I–IV)	5/9	29/46
	GVHD (chronic)	1/8	11/33
Kebriaei et al, 2009 [21]	Death (fall resulting in intracranial bleed)	1/31	-
	Death (GVHD)	4/31	-
	Death (infection)	3/31	-
	Death (cancer relapse)	1/31	-
	Infections (CMV viremia, adenovirus, bacteremia, Pseudomonal pneumonia, Enterococcal meningitis)	15/31	-
	Infusional toxicity	0/31	
	Tumour/malignancy (ectopic tissue)	0/31	
Ringden et al, 2006 [27]	Organ dysfunction (pulmonary - bronchiolitis obliterans)	1/8	
	Organ dysfunction (hepatic - increasing bilirubin)	1/8	-
	Infection	2/8	-
	Death (all cause)	4/8	13/16
	Death (infection)	2/8	-
	Death (multiorgan failure)	1/8	-
	GVHD (reoccurrence)	1/8	-
Lazarus et al, 2005 [24]	Infusional toxicity	0/46	-
	Death (cardiovascular)	1/46	-
	Death (gastrointestinal)	1/46	-
	Death (GVHD)	1/46	-
	Death (hemorrhage)	2/46	-
	Death (hepatic veno-occlusive disease)	1/46	-
	Death (infection)	2/46	-
	Death (relapse)	2/46	-
	Tumour/malignancy (ectopic tissue formation)	0/46	-
	Tumour/malignancy (relapse)	12/46	-
	GVHD (acute)	23/46	-
	GVHD (chronic)	22/46	-
Koç et al, 2002 [22]	Infusional toxicity (phlebitis)	1/11	-
	Fever (transient)	4/11	-
	Organ dysfunction (cardiovascular)	0/11	-
	Organ dysfunction (pulmonary)	0/11	-
	GVHD	0/11	-
Lazarus et al, 1995 [23]	Infusional toxicity (chest pain, shortness of breath, rash)	0/15	-
	Organ dysfunction (hematological)	0/15	-
	Organ dysfunction (hepatic)	0/15	-
	Organ dysfunction (neurological - neurological change)	0/15	-
	Organ dysfunction (pulmonary)	0/15	-
	Organ dysfunction (renal)	0/15	-
**Volunteer**			
Liu et al, 2006 [25]	Infusional toxicity (change in heart rate, respiration, oxygen saturation, blood pressure)	0/12	-
	Fever	0/12	-
	Organ dysfunction (cardiac)	0/12	-
	Organ dysfunction (hematological)	0/12	-
	Organ dysfunction (hepatic)	0/12	-
	Organ dysfunction (immune)	0/12	-
	Organ dysfunction (renal)	0/12	-
	Organ dysfunction (respiratory)	0/12	-

Abbreviations:

− = not reported, CMV = cytomegalovirus, GVHD = graft-versus-host disease,

**Table 7:** Frequency of adverse events in clinical trials with no control group.

Source	Statements of Safety and Adverse Events Reported	Frequency Treatment Group
**Cardiovascular**		
Yang et al, 2010 [44]	Infusional toxicity (anaphylaxis)	0/16
	Arrhythmias	0/16
	Organ dysfunction (cardiac)	0/16
	Infection	0/16
	Death	0/16
	Rehospitalization	0/16
**Neurological**		
Honmou et al, 2011 [32]	Infusional toxicity (pruritis at injection site, nausea)	1/12
	Fever	1/12
	Organ dysfunction (neurological)	0/12
	Infection (systemic)	0/12
	Tumour/malignancy	0/12
Karussis et al, 2010 [33]	Fever	21/34
	Organ dysfunction (pulmonary - dyspnea)	1/34
	Organ dysfunction (neurological: confusion, meningism, neck pain, leg pain, rigidity, difficulty walking or standing)	12/34
	Headache (related to lumbar puncture)	15/34
Zhang et al, 2008 [46]	Infusional toxicity	0/7
	Organ dysfunction (neurological - seizure (two episodes in same patient))	1/7
	Death	0/7
	Serious adverse events (cell related)	0/7
**Oncological/Hematological**		
Wang et al, 2011 [42]	Organ dysfunction (cardiac - myocarditis, hydropericardium causing heart failure)	2/10
	Infection (common cold)	1/10
	Death (cardiac, progression of disease)	2/10
	Disease relapse	3/10
Arima et al, 2010 [29]	Infusional toxicity	0/3
	Infection (pneumonia, no causative agent found)	2/3
	Death (GVHD)	2/3
	Death (infection)	1/3
Liang et al, 2010 [35]	Infusional toxicity	0/15
	Infection (systemic)	0/15
	Infection (upper respiratory tract)	‘some’/15
	GVHD	0/15
Sun et al, 2010 [41]	Death	0/16
Weng et al, 2010 [43]	Infusional toxicity	0/19
	Death (bronchiolitis obliterans from GVHD)	1/19
	Death (fungal infection)	2/19
	Death (malignancy relapse)	2/19
	Tumour/malignancy (relapse)	2/19
Zhang et al, 2010 [45]	Infusional toxicity	0/12
	Organ dysfunction (gastrointestinal - increased billirubin)	2/12
	Infection (CMV)	4/12
	Infection (bacterial and fungal pneumonia)	1/12
	Death (infection)	2/12
	Death (liver failure)	1/12
	Death (malignancy relapse)	2/12
	Tumour/malignancy (relapse)	4/12
	GVHD	4/12
Sun et al, 2009 [40]	Organ dysfunction (cardiovascular)	0/4
	Organ dysfunction (pulmonary)	0/4
	Infection	0/4
	Tumour/malignancy	0/4
	Metabolic dysfunction	0/4
Meuleman et al, 2009 [37]	Infusional toxicity	0/6
	Death (CMV reactivation	1/6
	Death (malignancy relapse)	1/6
	Infection (EBV reactivation, aspergillus)	3/6
	Tumour/malignancy (relapse)	1/6
Le Blanc et al, 2008 [34]	Infusional toxicity	0/55
	Infection (EBV)	3/55
	Death (relapse of original malignancy)	3/55
	Death (GVHD)	18/55
	Death (GVHD with concomitant infections (Aspergillosis, CMV, Enterococci, Klebsiella, E. coli))	16/18
	Death (AML de novo (patient had previous Pearson's disease))	1/55
	Death (multi-organ failure after severe hemorrhagic cystitis)	1/55
	Death (obstructive bronchiolitis and chronic GVHD)	1/55
	Tumour/malignancy (relapse)	3/55
Ringden et al, 2007 [39]	Death (infection)	4/10
	Death (multiorgan failure)	2/10
	Death (malignancy relapse)	1/10
	Transfusion of blood products	6/10
Fang et al, 2007 [31]	Death (fungal infection)	1/6
	Death (malignancy relapse)	1/6
	Tumour/malignancy (relapse)	1/6
**Gastrointestinal**		
Liang et al, 2011 [36]	Infusional toxicity	1/7
	Fever	1/7
	Organ dysfunction (gastrointestinal - relapse)	2/7
Duijvestein et al, 2010 [30]	Infusional toxicity (DMSO allergic reaction)	1/9
	Infusional toxicity (DMSO taste and smell)	9/9
	Infusional toxicity (headache)	3/9
	Fever	1/9
	Organ dysfunction (gastrointestinal – abdominal pain)	3/9
	Organ dysfunction (gastrointestinal – bloating)	1/9
	Organ dysfunction (gastrointestinal – diarrhea)	1/9
	Organ dysfunction (gastrointestinal – hemorrhoid)	1/9
	Organ dysfunction (gastrointestinal – nausea)	2/9
	Organ dysfunction (gastrointestinal – vomiting)	1/9
	Infection (common cold)	1/9
	Infection (acute otitis media)	1/9
	Fatigue, anorexia	4/9
	Worsening of disease	2/9
Mohamadnejad et al, 2007 [38]	Infusional toxicity	0/4
	Organ dysfunction (hepatic - volume decreased)	1/4
	Organ dysfunction (hepatic - total bilirubin increased)	2/4
	Tumour/malignancy (development of liver mass on follow-up CT scans)	0/4

Abbreviations:

− = not applicable; AML = acute myelogenous leukemia, CMV = cytomegalovirus, DMSO = dimethylsulfoxide, EBV = Epstein-Barr virus; GVHD = graft-versus-host disease.

**Table 8:** Descriptions and reporting of adverse events in randomized control trials.

Source	A Priori List of AE	A Priori Categorization of Serious and Non-serious AE	A Priori Listed Adverse Events	A Priori Description of Follow-up Duration and Frequency for AE
**Cardiovascular**				
Hare et at, 2009 [13]	Y	Y	Infusional toxicity (desaturation)¥	limited
			Arrhythmia (including nonsustained ventricular tachycardia, premature ventricular contractions)¥	
			Organ dysfunction (cardiac, gastrointestinal and renal, immune)+	
			Infection+	
			Death+	
			Tumour/Malignancy (ectopic tissue formation)	
			General disorders and administration site conditions (chest pain, fatigue)+	
			Rehospitalization+	
Chen et al, 2006 [11]	Y	N	Infusional toxicity (pulmonary edema)+	limited
			Arrhythmia+	
			Death+	
Wang et al, 2006 [17]	N	N	Infusional toxicity (anaphylaxis, embolism)+	N
			Fever+	
			Arrhythmia+	
			Organ dysfunction (cardiac)+	
			Death (cardiac)+	
Chen et al, 2004	Y	N	Arrhythmia¥Death+	limited
**Neurological**				
Lee et al, 2010 [14]	Y	Y	Immediate reactions:	limited
			Infusional toxicity (anaphylaxis)	
			Fever	
			Infection (systemic)	
			Organ dysfunction (cardiac+, renal, hepatic)	
			Local complications (hematoma, local infection at the site of bone marrow aspiration)	
			Vascular obstruction (tachypnea, oliguria, peripheral vascular insufficiency, recurrent stroke+)	
			Late:	
			Arrhythmia¥	
			Organ dysfunction (neurological - neuropsychological illness)+	
			Organ dysfunction (neurological - seizure)¥	
			Tumour/malignancy¥	
			Zoonoses from FBS use¥ (myoclonus, rapidly progressive dementia or ataxia)¥	
			Death (all cause mortality)¥	
Lee et al, 2008 [15]	Y	N	Immediate:	limited
			Infusional toxicity (anaphylaxis)	
			Fever	
			Infection (systemic)	
			Organ dysfunction (hepatic, renal)	
			Ischemic lesions or angiographic complications¥	
			Local complications (hematoma, local infection at site of bone marrow aspiration)	
			Vascular obstruction (tachypnea, oliguria, peripheral vascular insufficiency, stroke)	
			Late:	
			Death	
			Tumour/malignancy	
			Cholecystitis+	
Xie et al, 2007 [18]	N	N	Fever+	limited
			Death+	
			Headache+	
**Hematological/Oncological**				
Ning et al, 2008 [16]	Y	N	Infusional toxicity+	limited
			Infection+	
			Death+	
			Tumour/malignancy (relapse)*	
			GVHD*	

Abbreviations:

+ = adverse event not defined a priori,

* = clinical endpoint defined a priori,

¥ = both follow-up duration and frequency defined a priori for adverse event, AE = adverse event, FBS = fetal bovine serum, GVHD = graft-versus-host disease.

**Table 9:** Descriptions and reporting of adverse events in non-randomized controlled trials.

Source	A Priori List of AE	A Priori Categorization of Serious and Non-serious AE	A Priori Listed Adverse Events	A Priori Description of Follow-up Duration and Frequency for AE
**Cardiovascular**				
Mohyeddin-Bonab et al, 2007 [26]	N	N	Death+	N
**Hematological/Oncological**				
Vanikar et al, 2011 [28]	Y	N	Fever¥	limited
			Organ dysfunction (gastrointestinal, renal)¥	
			Infection+	
			Death+	
			GVHD¥	
			Transplant rejection*	
Baron et al, 2010 [19]	Y	N	Death (non-relapse mortality,+ all cause+)	limited
			Tumour/malignancy (relapse)¥	
			GVHD*	
Gonzalo-Daganzo et al, 2009 [20]	Y	Y	Infusional toxicity	N
			Death	
			Tumour/malignancy (relapse)	
			Adverse events evaluated using the National Cancer Institute Common Toxicity Criteria v.2	
			GVHD*	
Kebriaei et al, 2009 [21]	Y	N	Infusional toxicity	N
			Infection	
			Death+	
			Tumour/malignancy (formation of ectopic tissue, relapse)	
Ringden et al, 2006 [27]	N	N	Organ dysfunction (gastrointestinal, pulmonary)	N
			Infection+	
			Death+	
			GVHD*	
Lazarus et al, 2005 [24]	Y	Y	Infusional toxicity	limited
			Death (non-relapse mortality)	
			Tumour/malignancy (relapse, ectopic tissue)	
			Adverse events evaluated using the National Cancer Institute Common Toxicity Criteria v. 2	
			GVHD	
Koç et al, 2002 [22]	Y	N	Infusional toxicity (change in cardiovascular or respiratory status, hypersensitivity)¥	limited
			Fever¥	
			Organ dysfunction (cardiovascular, pulmonary)¥	
			GVHD+	
Lazarus et al, 1995 [23]	Y	N	Infusional toxicity (chest pain, shortness of breath, rash)+	limited
			Organ dysfunction (hematological, hepatic, pulmonary, renal)¥	
			Organ dysfunction (neurological)+	
**Volunteer**				
Liu et al, 2006 [25]	Y	N	Infusional toxicity (change in heart rate, respiration, oxygen saturation, blood pressure)¥	Y
			Fever¥	
			Organ dysfunction (cardiac, hematological, hepatic, immune, renal, respiratory)¥	

Abbreviations:

− = not reported,

+ = adverse event not defined a priori,

* = clinical endpoint defined a priori,

¥ = both follow-up duration and frequency defined a priori, AE = adverse event, GVHD = graft-versus-host disease,

**Table 10:** Descriptions and reporting of adverse events in clinical trials with no control group.

Source	A Priori List of AE	A Priori Categorization of Serious and Non-serious AE	A Priori Listed Adverse Events	A Priori Description of Follow-up Duration and Frequency for AE
**Cardiovascular**				
Yang et al, 2010 [44]	Y	N	Infusional toxicity (anaphylaxis)+	limited
			Arrhythmias¥	
			Organ dysfunction (cardiac)¥	
			Infection+	
			Death+	
			Rehospitalization+	
**Neurological**				
Honmou et al, 2011 [32]	Y	N	Infusional toxicity (pulmonary dysfunction, cardiac dysfunction)¥	limited
			Fever¥	
			Organ dysfunction (neurological)¥	
			Infection+	
			Tumour/malignancy¥	
Karussis et al, 2010 [33]	N	N	Fever+	N
			Organ dysfunction (neurological, pulmonary)+	
			Headache+	
Zhang et al, 2008 [46]	Y	Y	Adverse events evaluated using the National Cancer Institute Common Terminology Criteria for Adverse Events v.3	N
**Hematological/Oncological**				
Wang et al, 2011 [42]	Y	N	Organ dysfunction (cardiac)	limited
			Infection+	
			Death¥	
			Disease relapse¥	
Arima et al, 2010 [29]	N	N	Infusional toxicity+	N
			Infection (pneumonia)+	
			Death+	
Liang et al, 2010 [35]	N	N	Infusional toxicity+	N
			Infection (systemic, upper respiratory tract)+	
			GVHD+	
Sun et al, 2010 [41]	N	N	Death+	N
Weng et al, 2010 [43]	N	N	Infusional toxicity+	N
			Death+	
			Tumour/malignancy (relapse)+	
Zhang et al, 2010 [45]	Y	N	Infusional toxicity+	limited++
			Organ dysfunction (gastrointestinal)+	
			Infection	
			Death	
			Tumour/malignancy (relapse)	
			GVHD	
			Hemorrhage	
Sun et al, 2009 [40]	N	N	Organ dysfunction (cardiovascular, pulmonary, renal function*)	N
			Infection+	
			Tumour//malignancy+	
			Metabolic dysfunction+	
Meuleman et al, 2009 [37]	Y	N	Infusional toxicity (heart rate, blood pressure, temperature, skin reactions)¥	limited
			Infection (cytomegalovirus, aspergillus)¥	
			Death+	
			Late toxic side effects+	
			Tumour/malignancy+	
Le Blanc et al, 2008 [34]	Y	N	Infusional toxicity+	N
			Infection+	
			Death	
			Tumour/malignancy+	
Ringden et al, 2007 [39]	N	N	Death+	N
			Transfusion+	
Fang et al, 2007 [31]	N	N	Death+	N
			Tumour/malignancy (relapse)+	
**Gastrointestinal**				
Liang et al, 2011 [36]	N	N	Infusional toxicity+	N
			Fever+	
			Organ dysfunction (GI, disease relapse)+	
Duijvestein et al, 2010 [30]	Y	N	Infusional toxicity (DMSO allergic reaction, DMSO taste/smell, headache)+	limited
			Fever+	
			Organ dysfunction (gastrointestinal– nausea,vomiting, diarrhea, anorexia, bloating, abdominal pain, hemorrhoid)+	
			Infection (common cold, acute otitis media)+	
			Fatigue+	
			Worsening of disease¥	
Mohamadnejad et al, 2007 [38]	Y	N	Infusional toxicity (hemodynamic instability)¥	Y
			Organ dysfunction (worsening hepatic, renal)¥	
			Tumour/malignancy (development of liver mass)¥	

Abbreviations:

¥ = both follow-up duration and frequency defined a priori for adverse event,

+ = adverse event not defined a priori,

* = clinical endpoint defined a priori,

++ = only follow-up duration listed for all events a priori, follow-up frequency not listed, AE = adverse event, DMSO = dimethylsulfoxide, FBS = fetal bovine serum, GVHD = graft-versus-host disease.

### Immediate Adverse Events: Acute Infusional Toxicity and Fever

**Figure 2:**
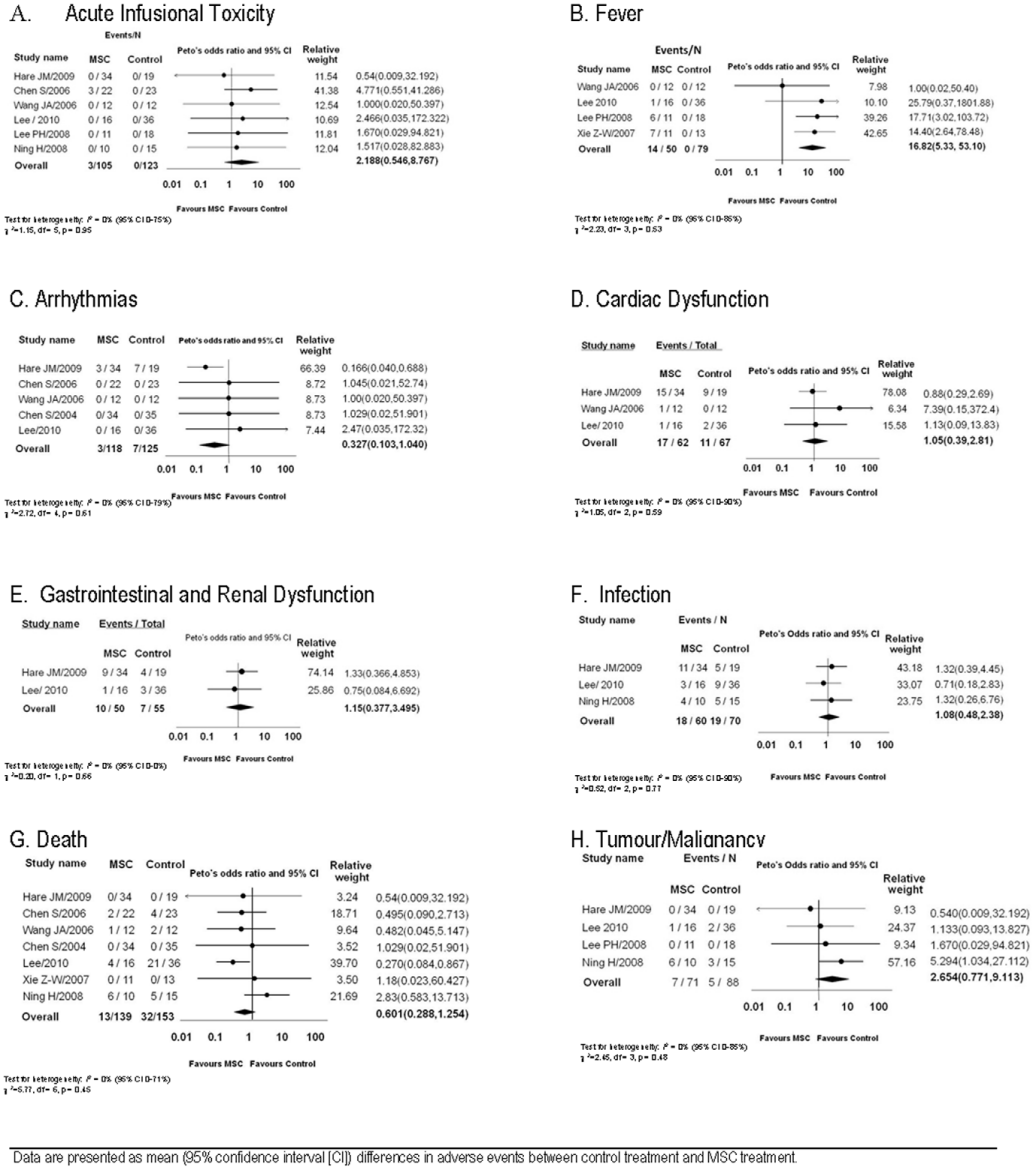
Confidence Intervals and Meta-analysis of Adverse Events. Data are presented as mean (95% confidence interval [CI]) differences in adverse events between control treatment and MSC treatment.

A meta-analysis of six RCTs revealed no significant differences in the occurrence of acute infusional toxicity between the MSC and control groups (OR 2.12, 95, 95% CI 0.55–8.77, Figure 2A). [11], [13]–[17] Six non-RCTs reported infusional toxicity; [20]–[25] one event occurred in 124 participants that received an MSC infusion (phlebitis during infusion [22]). Eleven uncontrolled clinical trials reported acute infusional toxicity which occurred in 12/159 (8%) participants. [29]–[32], [34], [36]–[38], [44]–[46]

Meta-analysis of the four RCTs revealed a significant increase in fever with MSCs as compared to the control group (OR 16.82, 95% CI 5.33–53.10, Figure 2B). [14], [15], [17], [18] All four studies used autologous MSCs. No non-RCTs reported fever. Four uncontrolled clinical trials reported fever in 24/62 (39%) participants. [30], [32], [33], [36] Three of these studies used autologous cells [30], [32], [33] and one used unmatched allogeneic cells. [36]

### Organ System Related Adverse Events

#### Cardiovascular Adverse Events

Meta-analysis of five RCTs that reported arrhythmias revealed no significant difference (OR 0.33, 95% CI 0.10–1.04, Figure 2C). [11]–[14], [17] No non-RCTs reported arrhythmias. One uncontrolled clinical trial that included 16 participants who received MSCs for acute myocardial infarction reported no arrhythmias. [44]

Three RCTs reported cardiac adverse events other than arrhythmias; a meta-analysis of these events revealed no difference between MSC and control treatment (OR 1.05, 95% CI 0.39–2.81, Figure 2D). [13], [14], [17] No non-RCTs reported cardiac adverse events. Three uncontrolled clinical trials reported cardiac adverse events in 2/30 (7%) participants. [40], [42], [44]

#### Gastrointestinal and Renal Adverse Events

Meta-analysis of pooled gastrointestinal and renal adverse events revealed no difference between MSC and control groups (OR 1.15, 95% CI 0.38–3.50), Figure 2E). One non-RCT reported gastrointestinal adverse events; 1/9 (11%) participants receiving MSCs had an elevated bilirubin, however rates in the control group were not reported. [27]

#### Pulmonary Adverse Events

One RCT reported pulmonary adverse events and described a significant improvement in pulmonary function with MSC treatment. [13] One non-RCT reported that 1/8 (13%) participants treated with MSCs developed bronchiolitis obliterans; rates of pulmonary adverse events were not reported for the control group. [27] Two uncontrolled clinical trials reported pulmonary dysfunction in 1/38 (3%) participants following MSC treatment. [33], [40]

#### Neurological Adverse Events

One RCT reported neurological dysfunction with seizures in 3/16 (19%) MSC treated participants and 5/36 (14%) of control participants. [14] None of the non-RCTs reported neurological dysfunction. Three uncontrolled clinical trials reported neurological dysfunction with events in 13/53 (25%) of participants. [32], [33], [46]

#### Hematological Adverse Events

None of the included studies reported hematological adverse events.

#### Infection Related Adverse Events

Meta-analysis of three RCTs that reported the occurrence of infection and revealed no differences between the MSC and control groups (OR 1.08, 95% CI 0.48–2.38, Figure 2F). [13], [14], [16] One non-RCT reported infection in 2/8 (25%) participants receiving MSCs; infection rates were not reported for the control group. [27] A second non-RCT reported death due to infection in 3/100 (3%) of participants receiving MSCs and 7/100 (7%) control participants. [28] Eight uncontrolled clinical trials reported infection which occurred in 5/91 (5%) participants. [30]–[32], [35], [40], [42]–[44]

#### Long Term Adverse Events: Death and Malignancy

Seven of eight RCTs reported death. A pooled analysis did not detect any differences in death between the MSCs and control group (OR: 0.60, 95% CI 0.28–1.25) (Figure 2G). [11]–[14], [16]–[18] Of the five non-RCTs, the total number of deaths in the MSC as compared to control group was 13/145 (9%) and 46/186 (25%) respectively. Eleven uncontrolled clinical trials reported death which was 50/160 (31%). [29], [31], [34], [37], [39], [41]–[46]

Meta-analysis of four RCTs that reported malignancy/tumour formation revealed no significant difference between MSC treated and control patients (OR 2.65, 95% CI 0.77–9.11, Figure 2H). [13]–[16] Two non-RCTs reported the occurrence of malignancy which was 7/29 (24%) and 10/62 (16%) in the MSC and control group respectively. [19], [20] Eight uncontrolled clinical trials reported malignancy/tumour formation; the occurrence was 11/118 (9%). [31], [32], [34], [37], [38], [40], [43], [45]

### Other Adverse Events

One RCT reported on prion disease over a five-year follow-up period and found none in the 16 participants given MSCs. No other studies monitored or reported on prion disease.

### Sensitivity Analyses

The small number of RCTs in each meta-analysis precluded the conduct of planned sensitivity analyses.

### Completeness of Reporting of Adverse Events

Twenty-eight of the 36 studies listed *a priori* at least one expected adverse event to be monitored, while the remainder did not. [17], [18], [26], [27], [29], [31], [33], [35] Five studies explicitly reported and separated serious from non-serious adverse events; [13], [14], [20], [24], [46] two of these referenced a standardized approach to detailing adverse events developed by other organizations. [20], [46] One study provided *a priori* a description of follow-up frequency and duration for all listed adverse events. [25] Eighteen studies provided this description for select adverse events, [11]–[16], [18], [19], [22]–[24], [28], [30], [32], [37], [42], [44], [45] Seventeen studies provided no details for follow-up duration and frequency of reporting of adverse events. [17], [20], [21], [26], [27], [29], [31], [33]–[36], [38]–[41], [43], [46]

## Discussion

This is the first systematic review and meta-analysis to comprehensively summarize the safety of systemic MSC administration. Our analysis was unable to detect associations between MSC treatment and the development of acute infusional toxicity, organ system complications, infection, death, or malignancy. There was, however, a significant association between MSC administration and transient fever. Our systematic review of non-RCTs supported these results. Six of seven RCTs and all non-RCTs described equal or fewer deaths with MSC treatment compared to control treatment. The completeness of adverse event reporting in the included studies was variable. However, aside from fever, the published current clinical trials suggest that the administration of MSCs is safe.

Although malignant transformation is a theoretical risk, our pooled analysis found no association between MSCs and tumour formation. Concerns related to tumourgenicity of MSCs were raised by preclinical studies demonstrating increased tumour burden in vivo. [47] Although recent position papers have suggested low probability of malignant transformation and tumour formation with MSCs, [7] our review is the first systematic analysis of the issue. Malignancy occurred only in studies involving participants with ongoing or previous malignancies; no de novo malignancies were observed.

We found no evidence of increased susceptibility to infection with MSC administration. Although MSC immunomodulatory effects may be beneficial in pro-inflammatory diseases, these same effects may leave a patient susceptible to infection. [48] In our review, infections were common in already immunosuppressed patients (e.g. following hematopoietic stem cell transplant), however the infection rates were similar to those previously published for similar populations. [49] In RCTs of participants without haematological malignancies, there were no differences between MSC and control participants. [13], [14]

There was a significant association between MSC administration and the development of fever. Fever was transient and not associated with long term sequelae. The mechanisms for fever are not clear but could be related to acute inflammatory reactions by a subset of patients to particular preparations of MSCs, not unlike similar reactions occasionally observed with red blood cell administration. [50]

Our review also addresses several issues and theoretical concerns with the cell product used in studies. First, concerns for immunogenicity may be unfounded as 13 studies used unmatched allogeneic MSCs with no reports of acute infusional toxicity. This supports the idea that MSCs are ‘immune-privileged’, a characteristic that may be explained by their low expression of MHC proteins and T-cell co-stimulatory molecules. [51] Second, the use of fetal bovine serum for culturing MSCs has been criticized for potentially introducing zoonotic contamination to the cell product (e.g. prion disease), and also potentially increasing the immunogenicity of the cells. [52], [53] Although the majority of included studies used fetal bovine serum, only one study specifically monitored for potential adverse events associated with its use. Concerns over fetal bovine serum will likely decrease in the future as expansion of MSCs in human blood products becomes more commonplace. The use of dimethylsulfoxide as a cryopreservative has been another potential concern with MSC therapy as this chemical is known to have toxic side effects and can cause hypersensitivity reactions. [54], [55] In our review only one study documented the occurrence of acute infusional toxicity and attributed it to dimethylsulfoxide. [30] A final concern is the viability of cells administered, as the administration of necrotic cells or cellular by-products may increase immunogenicity. Less than half of the studies included assessed and reported on viability of MSCs prior to infusion. Thus, greater vigilance may be needed in future studies for reporting cellular viability and monitoring for potential dimethylsulfoxide related adverse events.

No significant relationship between MSC administration and acute infusional toxicity was observed. The only RCT which described acute adverse reactions during infusion (acute and transient pulmonary edema in three participants) delivered MSCs to participants with chronic ischemic heart failure. [11] MSCs initially distribute to the lungs after intravascular administration; [56] thus, in susceptible patients this could cause a transient increase in pulmonary pressures and lead to pulmonary edema.

The reporting of adverse events was highly variable among the included studies. This may be related to editorial constraints of journals. Since use of MSCs may be associated with neoplastic growth long term, it is difficult to understand why approximately 50% of studies did not report follow up duration for adverse events. For highly experimental interventions with unestablished safety profiles, we contend that it is important to summarize the adverse reporting plan in the methods section of manuscripts and report short term and longer term events.

Our systematic review has several limitations. First, despite our comprehensive search strategy, there are a number of completed but unpublished industry sponsored studies and studies published in abstract form only that may alter the safety profile of MSCs. Second, we pooled adverse events across heterogeneous disease states. Given the limited number of clinical MSC studies, and the small sample sizes of each, it was important to pool data across trials to determine if any potential signals of harm existed. Previously, we have advocated this approach when individual trials are not adequately powered to detect potential harm. [57] However, we acknowledge that the occurrence, type, and severity of adverse events may vary significantly between different populations and according to different MSC characteristics (e.g. dose, type). The limited number of included RCTs precluded the conduct of these sensitivity analyses. Third, the majority of RCTs included in our analysis would be considered a high risk of bias. Although double blinding an MSC trial may be considered ethically unacceptable, it is difficult to justify the lack of concealment of the allocation of patients in many studies.

## Conclusions

Our study provides a systematic examination for adverse events related to the use of MSCs. We did not identify any significant safety signals other than transient fever. Results from our systematic review should provide some assurance to investigators and health regulators that, with the present evidence, this innovative therapy appears safe.

## Supporting Information

Appendix S1 Search strategy for medline, cochrane, and embase.
(DOCX)

Appendix S2 Search of clinicaltrials.gov.
(DOCX)

Appendix S3 Search and data extraction of abstracts from web of science.
(DOCX)
