# Supplementary material for: Safety of Cell Therapy with Mesenchymal Stromal Cells (SafeCell): A Systematic Review and Meta-Analysis of Clinical Trials
Source: PLoS One. 2012 Oct 25;7(10):e47559. doi: 10.1371/journal.pone.0047559 (PMC3485008; doi:10.1371/journal.pone.0047559)
Supplement: Appendix S1 — Search strategy for medline, cochrane, and embase. (DOCX) [file pone.0047559.s001.docx]

**Appendix s1. SEARCH STRATEGY for medline, cochrane, and embase.**

MEDLINE® In-Process & Other Non-Indexed Citations (Ovid)

MEDLINE® Daily (Ovid)

MEDLINE® 1950 to June 2011 (Ovid)

Cochrane Central Register of Controlled Trials 2^nd^ 2011 (EBM Reviews)

Cochrane Database of Systematic Reviews 2^nd^ Quarter 2011 (EBM Reviews)

1. mesenchymal stem cell*.mp. or exp Mesenchymal Stem Cells/
2. mesenchymal stem cell transplantation.mp. or exp Mesenchymal Stem Cell Transplantation/ or Bone Marrow Cells/
3. (mesenchymal adj (stem or stromal or progenitor or multipotent or bone marrow or adipose or placenta*)).mp.
4. Multipotent Stem Cells/ or Stromal Cells/ or multipotent stromal cell*.mp.
5. Mesoderm/cy [Cytology]
6. (ae or to or po or co).fs.
7. (safe or safety).ti,ab.
8. side effect$.ti,ab.
9. ((adverse or undesirable or harm* or serious or toxic) adj3 (effect* or reaction* or event* or outcome*)).ti,ab.
10. exp product surveillance, postmarketing/
11. exp adverse drug reaction reporting systems/
12. exp clinical trials, phase iv/
13. exp poisoning/
14. exp substance-related disorders/
15. exp drug toxicity/
16. exp abnormalities, drug induced/
17. exp drug monitoring/
18. exp drug hypersensitivity/
19. (toxicity or complication* or noxious or tolerability or hypersensitivity or abnormal*).ti,ab.
20. exp Postoperative Complications/
21. exp Intraoperative Complications/
22. randomized controlled trial.pt.
23. controlled clinical trial.pt.
24. randomized.ab.
25. placebo.ab.
26. drug therapy.fs.
27. randomly.ab.
28. trial.ab.
29. groups.ab.
30. (clinical trial* or multicenter study).pt.
31. or/22-30
32. (animals not (humans and animals)).sh.
33. 31 not 32
34. or/1-5
35. or/6-21
36. and/33-36

EMBASE 1980 to Week 21, 2011 (Ovid)

1. mesenchymal stem cell*.mp. or exp Mesenchymal Stem Cells/
2. mesenchymal stem cell transplantation.mp. or exp Mesenchymal Stem Cell Transplantation/ or Bone Marrow Cells/
3. (mesenchymal adj (stem or stromal or progenitor or multipotent or bone marrow or adipose or placenta*)).mp.
4. Multipotent Stem Cells/ or Stromal Cells/ or multipotent stromal cell*.mp.
5. Mesoderm/ [Cytology]
6. (ae or to or po or co).fs.
7. (safe or safety).ti,ab.
8. side effect.ti,ab.
9. ((adverse or undesirable or harm* or serious or toxic) adj3 (effect* or reaction* or event* or outcome*)).ti,ab.
10. exp adverse drug reaction/
11. exp drug toxicity/
12. exp intoxication/
13. exp drug safety/
14. exp drug monitoring/
15. exp drug hypersensitivity/
16. exp postmarketing surveillance/
17. exp drug surveillance program/
18. exp phase iv clinical trial/
19. (toxicity or complication* or noxious or tolerability or hypersensitivity or abnormal*).ti,ab.
20. exp postoperative complication/
21. exp Peroperative Complication/
22. Clinical trial/
23. Randomized Controlled Trial/
24. Randomization.mp. or exp Randomization/
25. Single blind procedure/
26. Double blind procedure/
27. Crossover procedure/
28. Placebo/
29. Randomi?ed controlled trial*.tw.
30. Rct.tw.
31. Random allocation.tw.
32. Randomly allocated.tw.
33. Allocated randomly.tw.
34. (allocated adj2 random).tw.
35. Single blind*.tw.
36. Double blind*.tw.
37. ((treble or triple) adj blind*).tw.
38. Placebo*.tw.
39. Prospective study/
40. Multicenter study/
41. or/22-40
42. Case study/
43. Case report.tw.
44. Abstract report/ or letter/
45. or/42-44
46. 41 not 45
47. or/1-5
48. or/6-21
49. and/46-48
