# Supplementary material for: Safety of Cell Therapy with Mesenchymal Stromal Cells (SafeCell): A Systematic Review and Meta-Analysis of Clinical Trials
Source: PLoS One. 2012 Oct 25;7(10):e47559. doi: 10.1371/journal.pone.0047559 (PMC3485008; doi:10.1371/journal.pone.0047559)
Supplement: Appendix S2 — Search of clinicaltrials.gov. (DOCX) [file pone.0047559.s002.docx]

**Appendix S2. SEARCH OF CLINICALTRIALS.GOV**

Search Strategy:

ClinicalTrials.gov (May 2010)

1. mesenchymal stem cell
2. mesenchymal stromal cell
3. multipotent stromal cell
4. bone marrow stem cell
5. adipose stem cell

Flow Diagram of Included and Excluded Studies from clinicaltrials.gov

**152** reports retrieved by initial search

**46** excluded due to unrelated topic

**106** reports reviewed

**93** excluded due to patient recruitment or incomplete data

**13** reports reviewed

2 pediatric participants

1 mononuclear cells

1 intraarticular injection

1 topical application

1 transdifferentiated MSCs

**7** final reports (all unpublished)

**Supplementaryappendix B. Unpublished studies from clinicaltrials.gov**

| **Study Name** | **URL** |
| --- | --- |
| Prochymal™ Adult Human Mesenchymal Stem Cells for Treatment of Moderate-to-Severe Crohn's Disease | [http://ClinicalTrials.gov/show/NCT00294112](http://clinicaltrials.gov/show/NCT00294112) |
| Safety and Efficacy Study of Adult Human Mesenchymal Stem Cells to Treat Acute GVHD. | http://ClinicalTrials.gov/show/NCT00136903 |
| Extended Evaluation of PROCHYMAL[tm] Adult Human Stem Cells for Treatment-Resistant Moderate-to-Severe Crohn's Disease | [http://ClinicalTrials.gov/show/NCT00543374](http://clinicaltrials.gov/show/NCT00543374) |
| Bone Marrow Stem Cells as a Source of Allogenic Hepatocyte Transplantation in Homozygous Familial Hypercholesterolemia | [http://ClinicalTrials.gov/show/NCT00515307](http://clinicaltrials.gov/show/NCT00515307) |
| Efficacy and Safety of Adult Human Mesenchymal Stem Cells to Treat Patients Who Have Failed to Respond to Steroid Treatment for Acute Graft Versus Host Disease (GVHD) | http://ClinicalTrials.gov/show/NCT00366145 |
| Safety and Efficacy of Prochymal for the Salvage of Treatment-Refractory Acute GVHD Patients | http://ClinicalTrials.gov/show/NCT00284986 |
| Stem Cell Therapy for Vasculogenesis in Patients With Severe Myocardial Ischemia | http://ClinicalTrials.gov/show/NCT00260338 |
