# Supplementary material for: Safety of Cell Therapy with Mesenchymal Stromal Cells (SafeCell): A Systematic Review and Meta-Analysis of Clinical Trials
Source: PLoS One. 2012 Oct 25;7(10):e47559. doi: 10.1371/journal.pone.0047559 (PMC3485008; doi:10.1371/journal.pone.0047559)
Supplement: Appendix S3 — Search and data extraction of abstracts from web of science. (DOCX) [file pone.0047559.s003.docx]

**Appendix s3. search strategy for web of science.**

Search Strategy:

**Web of Science September 2010**

Publication Name=(Blood) OR Publication Name=(Biology of Blood and Marrow Transplantation) OR Publication Name=(Bone Marrow Transplantation) OR Publication Name=(Cytotherapy) OR Publication Name=(Experimental Hematology) OR Publication Name=(Circulation) OR Publication Name=(Journal of the American College of Cardiology) OR Publication Name=(European Heart Journal) OR Publication Name=(Journal of Molecular and Cellular Cardiology) OR Publication Name=(Circulation) OR Publication Name=(Journal of Cardiac Failure) OR Publication Name=(European Respiratory Journal) OR Publication Name=(American Journal of Respiratory and Critical Care Medicine) OR Publication Name=(Chest) OR Publication Name=(Thorax) OR Publication Name=(Canadian Respiratory Journal) OR Publication Name=(Respirology) OR Publication Name=(International Journal of Tuberculosis and Lung Disease) OR Publication Name=(Neurology) OR Publication Name=(Annals of Neurology) OR Publication Name=(European Journal of Neurology) OR Publication Name=(European Journal of Neurology) OR Publication Name=(Multiple Sclerosis) OR Publication Name=(Critical Care Medicine) OR Publication Name=(Critical Care) OR Publication Name=(Gastroenterology) OR Publication Name=(Clinical Gastroenterology and Hepatology) OR Publication Name=(Canadian Journal of Gastroenterology) Publication Name =(Journal of Clinical Gastroenterology) Publication Name=(American Journal of Gastroenterology) OR Publication Name=(Nature Reviews Gastroenterology & Hepatology) Databases=SCI-EXPANDED, CPCI-S, CPCI-SSH Timespan=All Years

OR

Conference=((American Heart Association) OR (American College of Cardiology) OR (Transcatheter Cardiovascular Therapeutics) OR (European Society of Cardiology) OR (International Society for Heart Research) OR (World Congress of Cardiology) OR (Heart Failure Society of America) OR (American Society of Hematology) OR (American Society for Blood and Marrow) OR (European Bone Marrow Transplant) OR (International Society of Cellular Therapy) OR (International Society for Experimental Hematology) OR (European Respiratory Society) OR (American Thoracic Society) OR (American College Chest Physicians) OR (British Thoracic Society) OR (Canadian Respiratory Conference) OR (Asia-Pacific Society of Respirology) OR (International Union against Tuberculosis and Lung Disease) OR (Aspen Lung Conference) OR (American Academy of Neurology) OR (American Association of Neurologists) OR (European Federation of Neurological Societies) OR (World Federation of Neurology) OR (European Committee for Treatment and Research in Multiple Sclerosis) OR (Society of Critical Care Medicine) OR (International Symposium on Intensive Care and Emergency Medicine) OR Conference=(american gastroenterology association) OR OR Conference=(American College of Gastroenterology) Conference=(World Congress of Gastroenterology) OR Conference=(WGO) OR Conference=(Canadian Association of Gastroenterology)

AND

Topic=((Mesenchymal) OR (Multipotent Stem Cell*) OR (Stromal Cell*))

Databases=SCI-EXPANDED, CPCI-S, CPCI-SSH Timespan=All Years

**Appendix C. Flow Diagram of Included and Excluded Abstracts from Web of Science**

**1747** reports retrieved by initial search

**34** deduplicated by title

**1713** reports reviewed by title

**93** excluded due to patient recruitment or incomplete data

6 case reports

6 only pediatric patients

3 intrathecal MSC only

2 co-transplantation with other cell types

55 animal studies

9 in vitro studies

1 already included in review

6 duplicate abstracts

3 reviews

**168** reports reviewed by abstract

Data extracted from **24 abstracts:**

**5 full published reports retrieved and included in review**

**19 reports with abstract only**

**supplementary appendix c - Table 1A. Characteristics of randomized control trials**

| **Source** | **Country** | **Patient Population (n)** | **Intervention**  **(Source, Route)** | **Comparison** | **Patients Evaluated**  **(n (%male))**  **T C** | | **Age**  **(yrs±SD)**  **T C** | | **Follow Up Duration**  **(mo)** |
| --- | --- | --- | --- | --- | --- | --- | --- | --- | --- |
| **Cardiovascular** |  |  |  |  |  |  |  |  |  |
| Dib et al, 2009[1] | USA | ischemic and non-ischemic heart failure (20) | allogeneic, EC | mock mapping and injection | - | - | - | - | 3 |
| Zverev O et al, 2006[2] | RUS | non-acute ischemic heart disease (69) | - , IC | bone marrow mononuclear cells or contrast media alone | 18 | bone marrow mononuclear cells: 38  contrast media: 13 | - | - | 12 |
| **Oncological/Hematological** | | |  |  |  |  |  |  |  |
| Liu et al, 2008[3] | PRC | stem cell transplantation for leukemia (29) | allogeneic, IV | stem cell transplantation alone | 14 | 15 | - | - | - |

Abbreviations:

- = not reported, C = control group, EC= transendocardial, IC =intracoronary, IV =  intravenous, PRC = People’s Republic of China, RUS = Russia, T= treatment group, USA = United States of America

1. median (range)

**supplementary appendix c - Table 1B. Descriptions and Reporting of Adverse Events in randomized control trials**

| **Source** | **A Priori List of AE** | **A Priori Categorization of Serious and Non-serious AE** | **Potential Adverse Events Listed and/or Documented** | **A Priori Description of Follow-up Duration and Frequency for AE** |
| --- | --- | --- | --- | --- |
| **Cardiovascular** | | | | |
| Dib et al, 2009[1] | Y | N | Arrhythmia^+^  Organ dysfunction (cardiac*)  Death  Immune response (acute and subacute)  Procedural complications | limited |
| Zverev O et al, 2006[2] | N | N | Arrhythmia^+^  Organ dysfunction (cardiac*)  Death^+^ | limited |
| **Oncological/Hematological** | | | | |
| Liu et al, 2008[3] | N | N | Infusional toxicity^+^  Death^+^  Graft rejection^*^  GVHD^*^ | N |

Abbreviations:

+ = adverse event not defined a priori, * = clinical endpoint defined a priori, GVHD = graft-versus-host disease

**supplementary APPENDIX C - Table 1C. frequency of Adverse Events in randomized control trials**

| **Source** | **Statements of Safety and Adverse Events Reported** | **Frequency**  **Treatment Group** | **Frequency**  **Control Group** |
| --- | --- | --- | --- |
| **Cardiovascular** | | | |
| Dib et al, 2009[1] | Arrhythmias | 0/- | 0/- |
|  | Organ dysfunction (cardiac) | ‘improved’ | ‘decreased’ |
|  | Death | 0/- | 0/- |
|  | Immune response (acute or subacute) | NS | NS |
|  | Procedural complications | 0/- | 0/- |
| Zverev O et al, 2006[2] | Arrhythmia | 0/18 | - |
|  | Organ dysfunction (cardiac) | ‘improved’ | - |
|  | Death | 0/18 | - |
| **Oncological/Hematological** | | | |
| Liu et al, 2008[3] | Infusional toxicity | 0/14 | - |
|  | Death | - | 3/15 |
|  | Graft rejection | - | 1/15 |
|  | GVHD | 7/14 | 9/15 |

Abbreviations:

- = not reported, GVHD = graft versus host disease, NS = non-significantly different between treatment and control group

**supplementary APPENDIX C - Table 2A. Characteristics of NON RANDOMIZED control trial included**

| **Source** | **Country** | **Patient Population (n)** | **Study Design** | **Intervention**  **(Source, Route)** | **Comparison** | **Patients Evaluated**  **(n (%male))**  **T C** | | **Age**  **(yrs±SD)**  **T C** | | **Follow Up Duration**  **(mo)** |
| --- | --- | --- | --- | --- | --- | --- | --- | --- | --- | --- |
| **Cardiovascular** |  |  |  |  |  |  |  |  |  |  |
| Chen et al, 2009[4] | PRC | acute myocardial infarction (119) | cohort | autologous, IC | saline IC | 60 | 59 | - | - | 60 |

Abbreviations:

- = not reported, C = control group, IC = intracoronary, PRC = People’s Republic of China, T= treatment group

**supplementary appendix c - Table 2B. Descriptions and Reporting of Adverse Events in non randomized control trial**

| **Source** | **A Priori List of AE** | **A Priori Categorization of Serious and Non-serious AE** | **Potential Adverse Events Listed and/or Documented** | **A Priori Description of Follow-up Duration and Frequency for AE** |
| --- | --- | --- | --- | --- |
| **Cardiovascular** | | | | |
| Chen et al, 2009[4] | Y | Y | Organ dysfunction (cardiac^*^)  Death  Survival free from rehospitalization  Myocardial infarction  Target vessel revascularization | limited |

Abbreviations:

* = clinical endpoint defined a priori

**supplementary appendix c - Table 2C. frequency of Adverse Events in non randomized control trial**

| **Source** | **Statements of Safety and Adverse Events Reported** | **Frequency**  **Treatment Group** | **Frequency**  **Control Group** |
| --- | --- | --- | --- |
| **Cardiovascular** | | | |
| Chen et at, 2009[4] | Death | 2/60 | 1/59 |
|  | Rehospitalization | 6/60 | 12/59 |

**supplementary Appendix C - Table 3A. Characteristics of UNCONTROLLED TRIALS**

| **Source** | **Country** | **Patient Population (n)** | **Intervention**  **(Source, Route)** | **Patients Evaluated**  **(n (%male))** | **Age**  **(yrs±SD)** | **Follow Up Duration**  **(mo)** |
| --- | --- | --- | --- | --- | --- | --- |
| **Cardiovascular** |  |  |  |  |  |  |
| Nagaya N et al, 2007[5] | JPN | heart failure (11) | autologous, TE | 11 | - | 2 |
| Goncalvesova E et al, 2006[6] | SLO | ischemic cardiomyopathy (10) | autologous, IC | 10 (100) | 50±5 | 15±6^¥^ |
| **Neurological** | | |  |  |  |  |
| Bisaga G et al, 2009[7] | RUS | ALS, MS (10) | autologous, IV | 10 (54) | 30-63^*^ | 3-20* |
| Cho SC et al, 2009[8] | PRC, USA | ALS (22) | allogeneic, IV + IT | 22 | - | - |
| **Oncological/Hematological** | | |  |  |  |  |
| Introna M et al, 2009[9] | ITL | refractory GVHD (8) | allogeneic, IV | 8 | - | - |
| Koslov VA et al, 2009[10] | RUS | stem cell transplantation for malignancy (39) | autologous, IV | 39 | - | - |
| Resnick IB et al, 2009[11] | ISR | refractory GVHD (25) | -, IV, IHA^**^ | 25 | - | 20 |
| Stankevich Y, 2008[12] | RUS | stem cell transplant for malignancy (8)  refractory GVHD (9) | allogeneic, IV | 17 | - | 24 |
| von Bonin M et al, 2008[13] | GER | refractory GVHD (10) | allogeneic, IV | 10 | - | 191 (60-326)^+^ |
| Langelaan K et al, 2007[14] | NL | refractory GVHD (4) | allogeneic, IV | 4 | - | - |
| Lee J-H et al, 2007[15] | PRC | refractory GVHD (5) | allogeneic, IV | 5 (60) | 27-48^*^ | - |
| Zhang X et al, 2007[16] | PRC | stem cell transplantation for malignancy (12) | allogeneic, IV | 12 | - | 29-57^*^ |
| Ai H-S et al, 2005[17] | PRC | relapsed or refractory leukemia (11) | allogeneic, IV | 11 | 8-33^*^ | 3-18^*^ |
| Kim J-H et al, 2002[18] | ROK | stem cell transplantation for malignancy (12) | allogeneic, IV | 12 (50) | 29 (4-50)^+^ | - |
| **Gastrointestinal** | | |  |  |  |  |
| Onken et al, 2008[19] | USA | Crohn’s disease (10) | allogeneic, IV | 8 | - | 12 |

Abbreviations:

- = not reported, ALS = amyotrophic lateral sclerosis, GER = Germany, GVHD = graft-versus-host disease, IC = intracoronary, IHA = intrahepatic artery, ITL = Italy, ISR = Israel, IV =  intravenous, IT = intrathecal, JPN = Japan, MS = multiple sclerosis, NL = Netherlands, PRC = People’s Republic of China, ROK = Republic of Korea, RUS = Russia, SD = standard deviation, SLO = Slovakia, USA = United States of America, TE = transendocardial

* = range, + = mean (range), ¥ = mean ± SD

**one patient administered cells via intrahepatic artery

**supplementary APPENDIX C - Table 3B. Descriptions and Reporting of Adverse Events in UNCONTROLLED TRIALS**

| **Source** | **A Priori List of AE** | **A Priori Categorization of Serious and Non-serious AE** | **Potential Adverse Events Listed and/or Documented** | **A Priori Description of Follow-up Duration and Frequency for AE** |
| --- | --- | --- | --- | --- |
| **Cardiovascular** | | | | |
| Nagaya N et al, 2007[5] | N | N | Arrhythmias^+^  Organ dysfunction (cardiac^+^)  Tumour/malignancy^+^ | N |
| Goncalvesova E et al, 2006[6] | N | N | Arrhythmias^+^  Organ dysfunction (cardiac^*^)  Death^+^ | limited |
| **Neurological** | | | | |
| Bisaga G et al, 2009[7] | N | N | Acute or late AE^+^  Fever^+^  Death^+^  Headache^+^  Progression of disease^*^ | N |
| Cho SC et al, 2009[8] | Y | N | Organ dysfunction (hepatic, renal, muscle)  Cellular and humoral immune response | N |
| **Oncological/Hematological** | | | | |
| Introna M et al, 2009[9] | N | N | Acute or late AE (WHO common criteria)^+^  Death^+^ | N |
| Koslov VA et al, 2009[10] | N | N | Infusional toxicity^+^ | N |
| Resnick IB et al, 2009[11] | N | N | Acute or late AE^+^  Death^+^ | N |
| Stankevich Y, 2008[12] | N | N | Infusional toxicity^+^  Death^+^  GVHD^*^ | N |
| von Bonin M et al, 2008[13] | N | N | Death^+^ | N |
| Langelaan K et al, 2007[14] | Y | N | Infusional toxicity^+^ (hypotension, abdominal cramps, dyspnea, fever)  GVHD (progression)^*^ | limited |
| Lee J-H et al, 2007[15] | N | N | Infusional toxicity^+^  GVHD (progression)^*^ | N |
| Zhang X et al, 2007[16] | N | N | Infusional toxicity^+^  Infection^+^  Death^+^  GVHD^*^ | N |
| Ai H-S et al, 2005[17] | N | N | Death^+^  GVHD^*^ | limited |
| Kim J-H et al, 2002[18] | N | N | Infusional toxicity^+^  GVHD^*^  Graft rejection^+^ | N |
| **Gastrointestinal** | | | | |
| Onken et al, 2008[19] | N | N | Infection  Organ dysfunction (hematological)^+^  Tumour /malignancy  Crohns flare^+^ | limited |

Abbreviations:

+ = adverse event not defined a priori, * = clinical endpoint defined a priori, AE = adverse event, GVHD = graft-versus-host disease

**supplementary APPENDIX C - Table 3C. frequency of Adverse Events in uncontrolled trials**

| **Source** | **Statements of Safety and Adverse Events Reported** | **Frequency** |
| --- | --- | --- |
| **Cardiovascular** | | |
| Nagaya N et al, 2007[5] | Arrhythmias | 0/11 |
|  | Organ dysfunction (cardiac - pericardial effusion) | 0/11 |
|  | Ectopic tissue formation | 0/11 |
| Goncalvesova E et al, 2006[6] | Arrhythmias | 0/10 |
|  | Organ dysfunction (cardiac) | 0/10 |
|  | Death | 2/10 |
| **Neurological** | | |
| Bisaga G et al, 2009[7] | Acute or late AE (significant) | 0/13 |
|  | Fever | 2/13 |
|  | Death | 4/13 |
|  | Headache | 2/13 |
|  | Progression of disease | 3/13 |
| Cho SC et al, 2009[8] | Organ dysfunction – hepatic | 0/22 |
|  | Organ dysfunction – renal | 0/22 |
|  | Organ dysfunction – muscle breakdown | 0/22 |
|  | Cellular and humoral immune response | 0/22 |
| **Oncological/Hematological** | | |
| Introna M et al, 2009[9] | Acute of late AE | 0/8 |
|  | Death | 1/8 |
| Koslov VA et al, 2009[10] | Infusional toxicity | 0/39 |
| Resnick IB et al, 2009[11] | Acute or late AE | 0/25 |
|  | Death | 11/25 |
| Stankevich Y, 2008{Stankevich, 2008 #274 | Infusional toxicity | 0/17 |
|  | Death | 7/17 |
|  | GVHD | 0/8 |
| von Bonin M et al, 2008[13] | Death – liver failure | 3/10 |
|  | Death – alveolar hemmorhage | 1/10 |
|  | Death - microangiopathy | 1/10 |
| Langelaan K et al, 2007[14] | Infusional toxicity | - |
|  | GVHD (progression) | - |
| Lee J-H et al, 2007[15] | Infusional toxicity | 0/5 |
|  | GVHD (progression) | 3/5 |
| Zhang X et al, 2007[16] | Infusional toxicity | 0/12 |
|  | Infection (CMV) | 4/12 |
|  | Death | 5/12 |
|  | GVHD | 4/12 |
| Ai H-S et al, 2005[17] | Death | 3/11 |
|  | GVHD (acute) | 5/11 |
|  | GVHD (chronic) | 3/11 |
| Kim J-H et al, 2002[18] | Infusional toxicity | 0/12 |
|  | GVHD | 5/12 |
|  | Graft rejection | 0/12 |
| **Gastrointestinal** | | |
| Onken et al, 2008[19] | Infection | 2/8 |
|  | Organ dysfunction (hematological – anemia) | 1/8 |
|  | Tumour/malignancy | 0/8 |
|  | Crohn’s flare | 1/8 |

Abbreviations:

- = not reported, CMV = cytomegalovirus, GVHD = graft-versus-host disease

REFERENCES FOR SUPPLEMENTARY APPENDIX C:

1. Dib N, Henry T, Perin E, McCarthy M, Campbell A, et al. (2009) The First US Study to Assess the Feasibility and Safety of Endocardial Delivery of Allogenic Mesenchymal Precursor Cells in Subjects With Heart Failure: Three-Month Interim Analysis. American Journal of Cardiology 104: 77D-77D.

2. Zverev O, Boldueva S, Nemkov A, Shloydo E, Tsurupa S, et al. (2006) Improvement of cardiomyocyte function after transplantation of autologous bone marrow mesenchymal stem cells in patients with non-acute ischemic heart disease. European Heart Journal 27: 276-276.

3. Liu KY, Huang XJ, Chen YH, Chen H, Xu LP, et al. (2008) Cotransplantation of Mesenchymal Stem Cells Prompted Platelets Recovery in HLA-Haploidentical/Mismatched Blood and Marrow Transplantation: A Randomized Phase II Study. Blood 112: 1128-1128.

4. Chen SL, Tian NL, Liu ZZ (2009) Five-Year Clinical Outcomes after Transplantation of Autologous Bone Marrow Mesenchymal Stem Cells in Patients with Acute Myocardial Infarction Treated by Primary Stenting. American Journal of Cardiology 104: 74D-74D.

5. Nagaya N, Ohgushi H, Shimizu W, Yamagishi M, Noguchi T, et al. (2007) Clinical trial of autologous bone marrow mesenchymal stem cell transplantation for severe chronic heart failure. Circulation 116: 453-453.

6. Goncalvesova E, Lakota J, Fridrich V, Povinec P, Luknar M, et al. (2006) Feasibility and safety of intracoronary injection of mesenchymal stem cells in severe ischemic cardiomyopathy. Immediate, intermediate and long-term follow-up. European Heart Journal 27: 274-274.

7. Bisaga G, Odinak M, Novitsky A, Tirenko V, Sculyabin D, et al. (2009) Autologous multipotent mesenchymal stem cell transplantation in amyotrophic lateral sclerosis and multiple sclerosis: preliminary results of pilot study. Multiple Sclerosis 15: S139-S139.

8. Cho SC, Katzberg H, Kim BJ, Roh HJ, So YT (2009) Safety and Short-Term Effects of Umbilical Cord Mesenchymal Stem Cell Therapy in Amyotrophic Lateral Sclerosis: Review of Chinese Data. Neurology 72: A363-A363.

9. Introna M, Capelli C, Mico C, Algarotti A, Grassi A, et al. (2009) Human mesenchymal stromal cells expanded with human platelet lysate are safe and effective for the treatment of steroid-refractory graft-versus-host disease. Bone Marrow Transplantation 43: S133-S133.

10. Kozlov VA, Sergeevicheva VV, Lisukov IA, Kulagin AD, Kruchkova IV, et al. (2009) Cotransplantation of Mesenchymal Stromal Cells and Hematopoietic Stem Cells in Patients with Hematologic Malignancies. Experimental Hematology 37: S42-S43.

11. Resnick IB, Stepensky P, Shapira MY, Barkatz C, Elkin G, et al. (2009) Treatment of Severe Acute Graft-Versus-Host Disease with Mesenchymal Stromal Cells. Blood 114: 886-887.

12. Stankevich Y, Golovacheva A, Babenko E, Alyansky A, Paina O, et al. (2008) Prophylaxis and treatment of GvHD with mesenchymal stem cells in patients undergoing allo-HSCT. Bone Marrow Transplantation 41: S323-S323.

13. von Bonin M, Stoelzel F, Binder M, Goedecke A, Richter K, et al. (2008) Treatment of refractory acute GvHD with mesenchymal stem cells: organ-specific responses and outcomes. Bone Marrow Transplantation 41: S232-S233.

14. Langelaan K, Tick L, Willemze R, Roelofs H (2007) Bone marrow-derived mesenchymal stem cells for the treatment of steroid refractary acute graft-versus-host disease. Bone Marrow Transplantation 39: S273-S274.

15. Lee JH, Choi SJ, Seol M, Lee YS, Ryu SG, et al. (2007) Treatment of intractable graft-versus-host disease with bone marrow derived mesenchymal stem cells - A pilot clinical trial. Blood 110: 4974.

16. Zhang XY, Li JY, Cao KJ, Wu HX, Lu H, et al. (2007) Cotransplantation of HLA-identical mesenchymal stem cells and hematopoictic stem cells in chinese patients with hematologic diseases. Blood 110: 5061.

17. Ai HS, Guo M, Yu CL, Wang DH, Qiao JH, et al. (2005) Co-transplantation haploidentical mesenchymal stem cells and hematoietic stem cells with nonmyeloablative condition for treatment of relapsed or refractory acute leukaemia. Blood 106: 5439.

18. Kim JH, Jeong SH, Kang SY, Jeong JH, Park JS, et al. (2002) Co-transplantation of ex-vivo culture-expanded human mesenchymal stem cells and allogeneic hematopoietic stem cell transplantation - Report of 12 cases. Blood 100: 4234.

19. Onken J, Jaffe T, Custer L (2008) Long-term safety of prochymal adult mesenchymal stem cells in Crohn's disease. Gastroenterology 134: A661-A661.
